# Pectinmethylesterases (PME) and Pectinmethylesterase Inhibitors (PMEI) Enriched during Phloem Fiber Development in Flax (*Linum usitatissimum*)

**DOI:** 10.1371/journal.pone.0105386

**Published:** 2014-08-14

**Authors:** David Pinzon-Latorre, Michael K. Deyholos

**Affiliations:** Department of Biological Sciences, University of Alberta, Edmonton, Alberta, Canada; USDA-ARS-SRRC, United States of America

## Abstract

Flax phloem fibers achieve their length by intrusive-diffusive growth, which requires them to penetrate the extracellular matrix of adjacent cells. Fiber elongation therefore involves extensive remodelling of cell walls and middle lamellae, including modifying the degree and pattern of methylesterification of galacturonic acid (GalA) residues of pectin. Pectin methylesterases (PME) are important enzymes for fiber elongation as they mediate the demethylesterification of GalA *in muro*, in either a block-wise fashion or in a random fashion. Our objective was to identify PMEs and PMEIs that mediate phloem fiber elongation in flax. For this purpose, we measured transcript abundance of candidate genes at nine different stages of stem and fiber development and found sets of genes enriched during fiber elongation and maturation as well as during xylem development. We expressed one of the flax PMEIs in *E. coli* and demonstrated that it was able to inhibit most of the native PME activity in the upper portion of the flax stem. These results identify key genetic components of the intrusive growth process and define targets for fiber engineering and crop improvement.

## Introduction

Flax phloem fibers achieve their remarkable length through an extended period of intrusive growth. Intrusive elongation requires that fibers extend themselves through the middle lamellae of hundreds of cells, even destroying plasmodesmata in the process [1]. Once intrusive growth ceases, fibers begin to thicken their walls. The transition from elongation to thickening occurs around the snap point, a mechanically defined region of the stem described by Gorshkova and collaborators [2].

The demethylesterification of the cell wall plays a major role in the elongation and development of the phloem fibers of flax. Within the flax genome, 105 putative flax pectin methylesterases (LuPMEs) and 95 putative pectin methylesterase inhibitors (LuPMEIs) have been identified. The majority of these genes (77 LuPMEs and 83 LuPMEIs) have been demonstrated to be transcribed in at least one of the following tissues and developmental stages: floral buds, flowers, green capsules, early cortical peels, early fibers, late fibers, shoot apices, xylem, roots, leaf, senescent leaves [3]. Having thus defined the LuPME and LuPMEI families, we now have the opportunity to more precisely characterize these genes in the context of flax bast fiber development.

Heterologous expression is one tool that can be used to characterize gene function. PMEIs from different species have been successfully expressed in various systems. The mature proteins (i.e. without the signal peptide) of the Arabidopsis PMEIs AtPMEI-1 and AtPMEI-2 were both expressed in *Escherichia coli* strain Rosetta-gami (DE3) [4] and in *Pichia pastoris* strain X-33 [5] producing in both cases functional inhibitors. Also, the complete and mature protein of BoPMEI1, a PMEI from *Brassica oleracea*, was effectively expressed in *E. coli* strain ER2566. On the other hand, the heterologous expression of PMEs has produced less consistent results. The complete proteins of the type-2 PMEs QUARTET1 [6] and AtPME31 [7] were successfully expressed in *E. coli* strains BL21(DE3) and JM101, respectively. However, the mature portion (removing signal peptide and pro-region) of a type-1 PME (At1g11580) was expressed in *E. coli* strain M15 but was not functional compared to the native protein from Arabidopsis [8]. One explanation for these results is that post-translational modifications, such as glycosylation, may be necessary for the correct activity of some proteins as has been demonstrated for PMEs and PMEIs from kiwi fruit (*Actinidia chinensis*) [9], [10] and PMEs from mandarin orange (*Citrus sp.*) [11].

Our previous transcript profiling report of LuPMEs and LuPMEIs [3] did not provide sufficient spatial and temporal resolution to support efforts to identify PMEs and PMEIs that are involved in specific stages of fiber development. Consequently, to determine the expression of genes of interest at key developmental stages along the stem, we measured the activity of the PMEs, and assessed the transcript expression of 21 LuPMEs and 9 LuPMEIs in nine stages of fiber development. Our analysis allowed definition of a set of candidate PMEs and PMEIs with roles in fiber development during elongation, and during secondary cell wall deposition and maturation, and also a set of genes that could have important roles in xylem development.

## Materials and Methods

### Plant material

Plants were grown in a growth chamber at 22°C, with 16 hours day length, and were fertilized with 3 g/L of a 20–20–20 water soluble fertilizer (Plant-Prod) every two weeks. The soil was left to almost dry before watering the plants again.

Tissue was collected when plants reached between 46 and 48 cm, which occurred approximately five weeks after germination. At the time of harvest, the snap point was at an average distance of 7.1 cm from the shoot apex. In all cases, the leaves were removed. Sections 1-cm long were collected from positions along the stem as either whole stem, or as stem peels. Sections were collected at nine positions along the stem, based on the stage of development of the fiber [2], as follows: 0 to 1 cm (SA), 1 to 2 cm (1–2), 2 to 3 cm (2–3), 3 to 4 cm (3–4), 4 to 5 cm (A), 11.5 to 12.5 cm (B), 18 to 19 cm (C), 32 to 33 cm (D), and 44 to 45 cm (E). For microscopy, 100 µm cross sections for points A, B, C, D and E were obtained using a vibratome and 10 µm cross sections were obtained for position SA by wax-embedding in a Leica TP1020 tissue processor.

### RNA extraction

Fifteen 1 cm-fragments per tissue were used for the RNA extraction. The RNA was extracted using Trizol extraction coupled with the RNeasy Plant Mini Kit (QIAGEN). Tissue was ground, and 2 ml of Trizol (Sigma) were added, followed by incubation at 60°C for 5 min with vortexing. The supernatant was transferred to a new tube by centrifugation at 12000 rcf at 4°C for 15 min. 0.2 volumes of chloroform were added, mixed, and centrifuged at 12000 rcf at 4°C for 20 min. The supernatant was obtained, and from here the extraction was coupled with the RNeasy Plant Mini Kit. 0.25 volumes of solution RLT plus 0.5 volumes of cold ethanol were added. These were applied to RNeasy columns, and the kit manufacturer’s instructions were followed.

The RNA was tested for DNA contamination using a set of primers that flank an intron, producing differential product sizes for gDNA amplicons and for cDNA amplicons (Fw: 5′-TGCATATGCTCAGACCGACT-3′, Rv: 5′-TGGTGTAGATTTTCGGAAGAGAC-3). The RNA quality and concentration were assessed using the Agilent 2100 BioAnalyzer (Agilent Technologies, Inc.).

### cDNA synthesis and quantitative real time PCR

1 µg of RNA was used to synthesize cDNA using RevertAid H Minus Reverse Transcriptase (Thermo Scientific) and oligo(dT)18 primers following the manufacturer’s protocol.

The Applied Biosystems 7500 Fast Real-Time PCR System was used to conduct quantitative real time PCR (qRT-PCR) on the stem peel tissues, in 96 well-plates. For the whole stem tissues, we used the Applied Biosystems 7900 HT Fast Real-Time PCR System, in 384 well-plates. Three biological replicates and three technical replicates were used per sample. The cDNA was diluted 1∶40. The 10 µL sample mix consisted of 2.5 µL of diluted cDNA, 0.4 µM of each primer, and 1X MBSU buffer Tris (pH 8.3), containing KCl, MgCl_2_, Glycerol, Tween 20, DMSO, dNTPs, ROX as a normalizing dye, SYBR Green (Molecular Probes) as the detection dye, and an antibody-inhibited Taq polymerase. Primers used are the same used in Pinzon-Latorre and Deyholos [3].

### Gene clustering based on expression

The STEM (Short Time-series Expression Miner) software package [12], was used to cluster the genes according to their transcript expression patterns. The negative of the dCT values were used as input in STEM, which was run using the “normalize data” option, so the values of the first tissue were transformed to 0. We also used a minimum correlation of 0.8, with a maximum of nine model profiles for whole stem samples and five model profiles for stem peel samples, and also the minimum absolute expression change was adjusted to 2, so those genes in which there was less than a 4 fold difference between the highest and the lowest expression value were not used to generate the clustering.

### Heterologous expression

The coding region of the mature protein (i.e. excluding the signal peptide) of LuPMEI45 was used for heterologous expression. It was synthesized (Bio Basic Inc.) with codon optimization for *E. coli* (File S1) and was transformed into pET22b(+) (Novagen, Madison, WI, USA) via the restriction sites XhoI and NcoI. This plasmid was then transformed into *E. coli* Rosetta-Gami B(DE3)pLysS (Novagen, Madison, WI, USA). The empty pET22b(+) vector without inserts was used as a negative control in the various assays.

A single colony was grown overnight at 37°C in 2XYT medium plus chloramphenicol (34 µg/ml), tetracycline (12.5 µg/ml), kanamycin (15 µg/ml) and ampicillin (50 µg/ml). From this, 1 mL was transferred into 1 L of medium, and grown at 37°C until OD_600 nm_ 0.5–0.6, which was cooled on ice. IPTG at a final concentration of 1 mM was added, followed by growth for 18 hours at 20°C. Cells were pelleted at 4°C at 8000 rpm for 20 min. All subsequent manipulations were performed at 4°C unless otherwise indicated. The pellet was then mixed with 5% v/v of the original volume of 300 mM NaCl Tris HCl (the pH was at least one unit away from the predicted pI of the protein). This solution was left for at least 4 hours at −20°C, and was then sonicated at 55% for 30 seconds five times, with the intermediate tip of a Sonic Dismembrator model 300 (Fisher), with at least 1 min on ice between pulses. It was then centrifuged at 15000 rpm for 30 min at 4°C. The supernatant was incubated with 2% v/v of Ni-NTA agarose (QIAGEN) and rocked overnight prior to purification.

The His-tagged protein was purified using a Poly-Prep chromatography column (0.8×4 cm) which was prepared by adding 2 volumes of 50 mM Tris-HCl and 300 mM NaCl at the selected pH. The protein extract was then added, and it was washed with two volumes of 50 mM Tris-HCl, 1.5 M NaCl, then with 50 mM Tris-HCl, 300 mM NaCl, 20 mM imidazole, and then with 50 mM Tris-HCl, 300 mM NaCl, 40 mM imidazole. The protein was eluted with 5 ml of 50 mM Tris HCl, 1 M NaCl and 250 mM imidazole, containing one cOmplete ULTRA protease inhibitor (Roche) tablet per 10 ml. Five 1 ml-fractions were obtained, which were dialyzed against 50 mM Tris-HCl, 300 mM NaCl using a Amicon Ultra 3K centrifugal filter unit (Millipore).

### LC MS/MS

The protein observed with the expected size in the Coomassie-stained polyacrylamide gel was confirmed by in-gel tryptic digestion and identification by LC MS/MS analysis in the Institute for Biomolecular Design (University of Alberta).

### Protein extraction for PME activity

Proteins were extracted from three biological replicates according to the protocol of Hongo and collaborators [13]. Seven fragments (1 cm length each) obtained from equivalent positions along stems of different individuals were pooled for each extraction. Tissues were ground in liquid nitrogen, and 1 mL of extraction buffer, containing 12.5 mM citric acid, 50 mM phosphate buffer pH 7.0, with 1 M NaCl plus one tablet per 10 mL of cOmplete ULTRA protease inhibitor (Roche), was added. The sample was incubated at 4°C on a rocker and was then centrifuged at 15,000 rcf for 15 min, and the supernatant was collected. The protein concentration was determined using Qubit Fluorometric Quantitation (Life Technologies).

### Radial activity assay

The radial assay was done with three biological replicates and three technical replicates as described by Downie and collaborators [14], with modifications [13]. 2% (w/v) agarose was dissolved in McIlvaine buffer with pH adjusted to 6.0 and 7.0 and was autoclaved, after which 0.1% (w/v) of highly methylesterified pectin (Sigma-Aldrich, P9561) was added and dissolved. From this mixture, 13 mL was poured into 90 mm petri dishes. After cooling, wells with a diameter of 4 mm were punched in the agarose using a micropipette tip. 10 µL of freshly extracted protein (396 µg/mL) plus 10 µL of 50 mM Tris HCl 300 mM NaCl buffer were dispensed into each well. This was incubated for 18 h at 28°C, and the gel was stained with an aqueous solution of 0.05% (w/v) ruthenium red for 1 h and washed with distilled water. The plates were photographed immediately and the area of the halo was measured using ImageJ [15].

### PMEI inhibitory activity

The ability of recombinant PMEI to inhibit native PME activity in proteins extracted from flax stems was tested as in Raiola and collaborators [5]. For this purpose, PME activity was assayed as described above. For inhibition assays, 10 µL of flax cell wall proteins (396 µg/mL) were mixed with 10 µL of heterologous LuPMEI45 dialyzed solution (146 µg/mL) and incubated for 30 min at room temperature, and then the mixture was added to a well in the assay plate (20 µl per well).

## Results

### Tissues corresponding to the different stages of development

**Figure 1:**
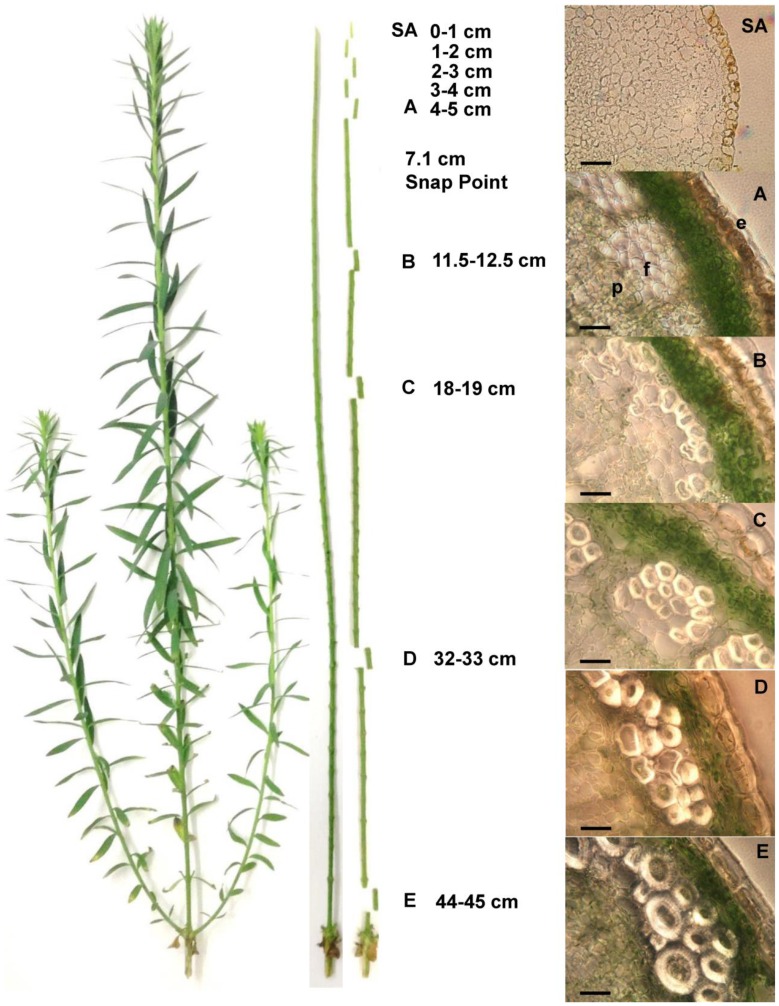
Location in the stem of the tissues used for the experiments used in this study. Plants were 5 weeks old, in vegetative stage, and their height from the hypocotyls was between 46 to 48 cm. Tissues were harvested and rapidly frozen with liquid nitrogen for RNA and protein extraction. 100 µm (points A to E) and 10 µm (point SA) cross sections. Bar: 50 µm. e: epidermis, f: fibers, p: phloem.

Gorshkova and collaborators [2] defined different stages of development of flax fibers relative to a mechanically defined “snap point” on the stem. In general, fiber specification and elongation occur apically to the snap point, and fiber cell wall thickening occurs basally. With this frame of reference, we examined the stem anatomy of linseed flax (variety CDC Bethune), in plants 46 to 48 cm long, ∼5 weeks after germination, just before flowering. Based on our observations, and with reference to the precedent established by Gorshkova, we identified nine positions along the stem that represented progressive stages of fiber development (Figure 1). Five of these positions (points SA to A) were apical to the snap point, and four positions (B through E) were basal to the snap point. A 1 cm segment of whole stem was harvested at each of the nine positions. Additional 1 cm segments were obtained from positions A through E, and these were peeled to obtain only the outer tissues of the stem (epidermis, cortex, phloem, and some cambial zone cells), while excluding xylem. The four, apical-most segments (SA to 3–4) were too delicate to effectively peel.

### Selection of candidate genes

To identify genes that affect the development and extractability of flax fibers, we selected 21 LuPMEs and 9 LuPMEIs for detailed characterization (Table S1). The selection of these genes was based on two previous studies: (i) previously published Fluidigm qRT-PCR expression data that showed the selected genes to be enriched in fiber-bearing tissues [3], and (ii) oligonucleotide microarray data that showed transcripts of the selected genes to be enriched in at least one of the points of the stem [16].

We tested three genes for their suitability as endogenous controls in the qRT-PCR assays. These three genes (GAPDH, ETIF1, ETIF5A) were selected for evaluation based on the results from Huis and collaborators [17]. We used BestKeeper software [18] to evaluate the expression stability of these genes in the tissues used in this study. ETIF1 had the least overall variation with a standard deviation of 0.67, followed by ETIF5A (0.74) and GAPDH (0.75). The best correlation between BestKeeper index and candidate reference gene was for ETIF5A (0.995), followed by GAPDH (0.992), and then ETIF1 (0.984). All three genes were therefore considered suitable as endogenous references for the qRT-PCR experiments described here, and the geometric mean of their Ct value was used to calculate the delta-C_T_.

**Figure 2:**
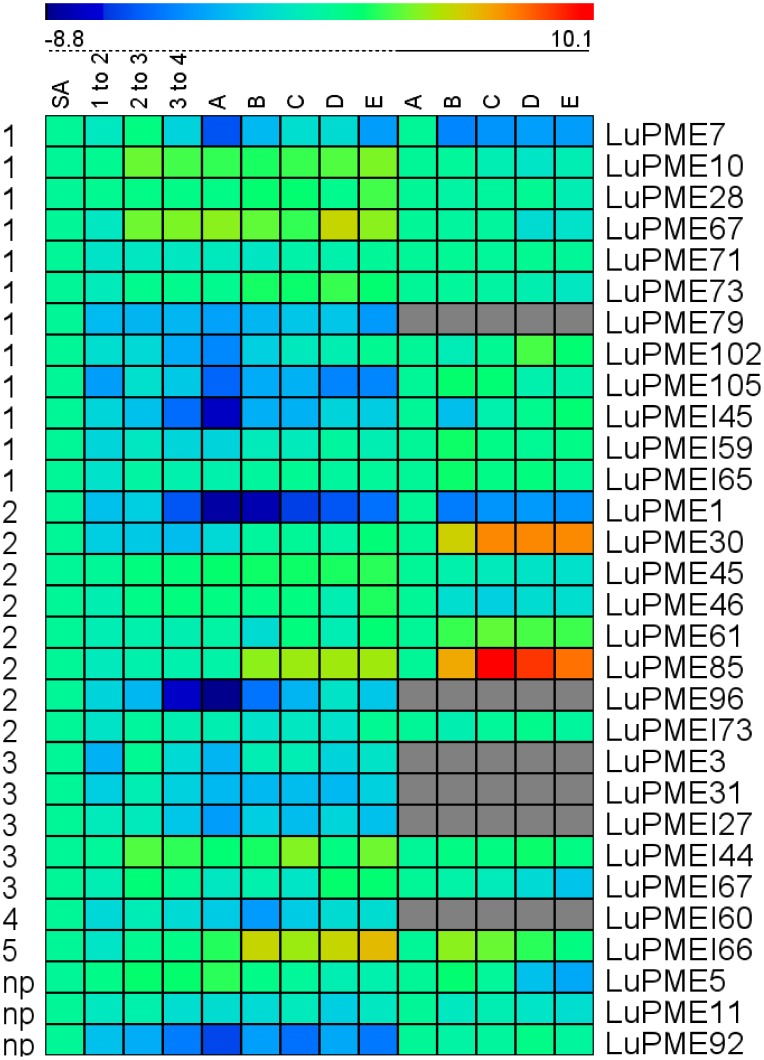
Transcript expression of genes from whole stem and stem peel tissues (ddCT). dCT was obtained by subtracting the geometric mean of the three endogenous controls used to the Ct value of the genes studied for every biological replicate. Here we show the average of the three biological replicates. The negative of the dCT value was used to calculate the ddCT, so a higher value represents higher transcript abundance. The ddCT was obtained by substracting the dCT value from the SA for whole stem tissues, and from point A, for stem peel tisues. The tissues below the dotted line are whole stem tissues, and below the solid line are stem peel tissues.

We measured relative transcript abundance of 21 LuPMEs and 9 LuPMEIs in fourteen stem segments and stem peels (Figure 2, and Figure S1). Transcripts of four genes (*LuPME3*, *LuPME96*, *LuPMEI27*, and *LuPMEI60*) could not be reliably detected in stem peel and these data were therefore not included in the results presented here. Because we were interested in identifying genes that were dynamically expressed during stem and fiber development, we calculated the maximum fold-change in transcript abundance between any two tissues (i.e. difference between the highest and the lowest mean dCT values (Table 1). We found 10 genes in the whole stem and 6 genes in the stem peel that differed at least 20-fold in transcript abundance between any two positions along the stem. Among them, the three highest fold-changes in the whole stem tissues were observed in *LuPME85* (419 fold higher at A compared to SA), *LuPME61* (307 fold higher at A respect to SA), and *LuPME1* (191 fold higher at A compared to segment 2–3). Among outer stem peels, the three genes with the greatest difference in transcript abundance were *LuPME79* (1085 fold higher at A respect to C), *LuPME67* (153 fold higher at A respect to C), and *LuPMEI66* (37 times higher at E respect to B).

**Table 1:** Tissue enrichment of selected PME and PMEIs.

	Highest fold change in whole stem					Highest fold change in stem peel				
Gene	Fold change	CI	Max at	Min at	p-value	Fold change	CI	Max at	Min at	p-value
**LuPME1**	191.4	97.8–374.6	A	2to3	****	20.3	6.2–66.3	B	A	****
**LuPME3**	13.4	4.8–36.9	E	SA	***					
**LuPME5**	3.4	2–5.6	1to2	C	***	4.9	3.5–6.6	A	B	**
**LuPME7**	6.5	5.1–8.3	SA	E	***	2.1	2–2.3	E	B	ns
**LuPME10**	15.7	9.4–26.1	SA	E	****	2.7	1.4–5.1	D	B	*
**LuPME11**	3	1.8–5	1to2	E	**	2.3	1.7–3.3	A	D	*
**LuPME28**	7.8	3.9–15.5	SA	D	***	2.3	1.6–3.4	E	B	*
**LuPME30**	45.1	33.4–61	A	E	****	9.2	6.3–13.4	A	D	****
**LuPME31**	40.2	3.1–527.3	A	SA	****	4.7	2.9–7.7	A	B	**
**LuPME45**	185.9	88–392.6	A	SA	****	19.5	12.9–29.4	B	E	****
**LuPME46**	4.6	2.9–7.3	A	D	*	5.2	3.6–7.4	A	B	**
**LuPME61**	307	171.5–549.4	A	SA	**	24.3	13.5–43.9	B	A	****
**LuPME67**	19.9	17.7–22.4	3to4	E	****	152.6	94.3–246.8	A	C	****
**LuPME71**	6	1.2–30.4	SA	E	**	3	1.2–7.8	C	A	**
**LuPME73**	7.9	5.1–12.3	B	E	***	11.8	10.9–12.7	A	C	****
**LuPME79**	26	7.3–92.3	1to2	E	****	1084.9	707.9–1662.6	A	C	****
**LuPME85**	419.4	110.4–1593.1	A	SA	****	31.5	22.5–44.2	B	E	****
**LuPME92**	3.4	1.7–6.6	D	E	***	2.6	2–3.4	A	D	*
**LuPME96**	15.8	7.8–32.3	1to2	2to3	****					
**LuPME102**	8.8	5.5–14	SA	E	****	2.2	0.8–6.1	A	D	ns
**LuPME105**	49.3	10.2–237.8	1to2	D	****	3.6	3–4.3	D	C	***
**LuPMEI27**	6.2	3.3–11.7	B	2to3	*					
**LuPMEI44**	11.8	6.8–20.5	A	SA	***	1.4	1–2.1	B	C	ns
**LuPMEI45**	17	7.1–40.8	SA	C	****	4.8	2.4–9.8	A	D	****
**LuPMEI59**	6.5	1.6–26.8	C	D	***	6.6	3.3–13.3	E	B	****
**LuPMEI60**	13.6	1.3–137.8	B	2to3	ns					
**LuPMEI65**	91.8	39–215.7	1to2	E	****	18.2	7.4–44.9	A	B	****
**LuPMEI66**	6.5	3.9–11	SA	A	****	37.4	21.1–66.4	E	B	****
**LuPMEI67**	4.4	3.5–5.4	D	2to3	ns	2	1.7–2.4	E	C	ns
**LuPMEI73**	74.7	43.9–127	A	SA	****	2.9	2.2–3.9	A	D	**

The fold-enrichment between the tissue sample with the highest transcript abundance and the lowest transcript abundance was calculated for each gene. This calculation was done separately for whole stem (WS) and stem peel (SP) samples. Fold enrichment is shown in a linear scale and is the mean of 3 measurements from 3 biologically independent samples. The p-value of the difference between the two points denoted was obtained by an ANOVA test that was followed by a Tukey’s multiple comparisons test using GraphPad Prism version 6.00 for Windows The asterisks denote the p-value as follows. *0.01–0.05; **0.001–0.01, ***0.0001–0.001; ****<0.0001. ns: non-significant difference (p>0.05). The values not shown are genes that were not detected in those tissues. The confidence interval (CI) was calculated by using one standard deviation of the difference of the dCT between the two tissues compared.

We also compared the expression of the genes in the same position in the whole stem and the stem peel (Tables 2, 3, and S2). Five genes (*LuPME67*, *LuPME79*, *LuPME92*, *LuPMEI45*, and *LuPMEI66*) showed an expression at least 20 times higher in at least one of the whole stem tissues in comparison to the corresponding position in the stem peel; all of these observations of differential expression were made in tissues below the snap point (B to E). Meanwhile, transcripts of four genes (*LuPME1*, *LuPME45*, *LuPME85*, and *LuPMEI65*) were at least 20 times more abundant in stem peel tissue than in a whole stem tissue. In all the cases the 20-fold change in expression was observed in tissues below the snap point.

**Table 2:** Tissue enrichment in whole stem compared to stem peels of genes with higher expression in whole stem.

	Fold change					CI of genes with higher expression in whole stem				
Gene/Point	A	B	C	D	E	A	B	C	D	E
LuPME1	3.8					1.6–9.3				
LuPME3										
LuPME5	1	3.6	1.8	2.6	1.6	0.7–1.5	2.6–4.9	1.2–2.6	1.6–4.3	0.9–2.6
LuPME7	1.3	1.6	1.2			1.1–1.5	0.7–3.7	0.9–1.5		
LuPME10	3.6	8.5	3.7	1.9	1.8	3–4.2	6–12.2	2.1–6.6	0.7–5	1.1–2.8
LuPME11	1.2	2.8	1.4	1.8		0.8–1.8	2.3–3.4	1.1–1.8	1.7–1.9	
LuPME28	1.4	1.3	1.2			0.9–2.2	1–1.5	0.9–1.6		
LuPME30	2.3					1.5–3.4				
LuPME31	1					0.1–9.1				
LuPME45	3.1					1.6–5.9				
LuPME46										
LuPME61	7.6					3.6–16.1				
LuPME67		5.7	17.8	22.9	9.5		5–6.4	13.4–23.7	14.5–36	6.5–14
LuPME71										
LuPME73		2.1		1.2			1.4–3.3		0.6–2.5	
LuPME79		2.1	21.8	8.6	4		1.5–2.9	13.9–34.3	4.2–17.4	1.0–16
LuPME85	5.8					1.8–18.8				
LuPME92	5.6	12.2	16.3	26.5	5.5	3.1–10.2	10.4–14.3	13.2–20.1	14–50	4.2–7.1
LuPME96										
LuPME102				1					0.7–1.5	
LuPME105	1.6	4.2	7.1		1	1.2–2.1	2.8–6.2	4.9–10.3		0.7–1.4
LuPMEI27										
LuPMEI44	4.2		1.9		1.5	2.8–6.1		1.5–2.4		1–2.4
LuPMEI45	9.1	21.3	6.9	56.4	8.3	5.1–16.1	9.3–49	3.6–13.2	26.5–119.9	3.1–22
LuPMEI59	13.7	12.7	15	1.2		9.6–19.6	5.4–29.8	6.9–32.3	0.3–3.9	
LuPMEI60										
LuPMEI65										
LuPMEI66	3.5	36.9	23.9	4.3	2.1	2.4–5.3	21.3–63.8	19.3–29.4	2.5–7.4	1.5–3
LuPMEI67	14.3	12.9	9.2	17.2	5.1	9.4–21.6	9.8–17	6.8–12.5	16.6–17.8	4.3–6.2
LuPMEI73	3		2.4	1	2	2.2–4.1		1.8–3.1	0.7–1.5	1.2–3.3

The fold-enrichment between the tissue sample with the highest transcript abundance and the lowest transcript abundance was calculated for each gene. This calculation was done separately for whole stem (WS) and stem peel (SP) samples. Fold enrichment is shown in a linear scale and is the mean of 3 measurements from 3 biologically independent samples. The values not shown are genes that were not detected in those tissues. The confidence interval (CI) was calculated by using one standard deviation of the difference of the dCT between the two tissues compared. The significance of the difference between the points in the stem in stem peel and whole stem is shown in Table S2.

**Table 3:** Tissue enrichment in whole stem compared to stem peels of genes with higher expression in stem peel.

	Fold change					Confidence Interval				
Gene/Point	A	B	C	D	E	A	B	C	D	E
LuPME1		53.2	121.2	86.3	15.2		20.6–137.2	99.1–148.1	22.1–337.2	9.5–24.4
LuPME3										
LuPME5										
LuPME7				1.4	1.7				1.1–1.7	1.5–2
LuPME10										
LuPME11					1					0.6–1.7
LuPME28				1.8	1.5				1–3.4	0.5–4.8
LuPME30		2.8	3.4	1.2	4.9		1.6–4.7	2.4–4.7	0.4–3.6	3.7–6.6
LuPME31		1	1.4	1.4	1.4		0.4–2.5	1.0–2.0	0.6–3.4	1–1.9
LuPME45		40.5	6.7	9.6	4.7		26.3–62.3	4.9–9.1	5.9–15.8	3–7.5
LuPME46	2.9	1.5	2.5	6.5	3.1	2–4.3	1.2–1.9	1.7–3.4	3.2–13.3	1.9–5
LuPME61		4	7	9.8	18.2		2.9–5.5	4.4–10.9	4.7–20.5	12.2–27.1
LuPME67	1.9					1.3–2.9				
LuPME71	4.9	8.3	15.4	3.3	17.1	1.7–14.1	2–33.9	3.6–66.2	1–11.6	3.5–85
LuPME73	10.8		2.1		3.4	9.3–12.6		1.6–2.7		2.8–4.1
LuPME79	3.2					1.6–6.4				
LuPME85		12.5	12.8	34.9	3.1		2.9–53.6	6.8–24.2	15.5–78.5	1.3–7.2
LuPME92										
LuPME96										
LuPME102	2.4	2.5	2		6.9	0.9–6.7	0.9–7.2	0.9–4.5		4.4–10.8
LuPME105				2.7					1.3–5.7	
LuPMEI27										
LuPMEI44		1.3		1.4			0.8–2		0.9–2.2	
LuPMEI45										
LuPMEI59					1.7					1.4–2.1
LuPMEI60										
LuPMEI65	16.6	5.9	4.5	15.7	56.1	11.2–24.4	2.3–14.7	1.4–14.7	6.7–37.1	25.4–123.7
LuPMEI66										
LuPMEI67										
LuPMEI73		1.4					1.2–1.6			

The fold-enrichment between the tissue sample with the highest transcript abundance and the lowest transcript abundance was calculated for each gene. This calculation was done separately for whole stem (WS) and stem peel (SP) samples. Fold enrichment is shown in a linear scale and is the mean of 3 measurements from 3 biologically independent samples. The values not shown are genes that were not detected in those tissues. The confidence interval (CI) was calculated by using one standard deviation of the difference of the dCT between the two tissues compared. The significance of the difference between the points in the stem in stem peel and whole stem is shown in Table S2.

### Clustering of transcript expression data

To identify shared patterns of transcript expression among the genes surveyed, we clustered the qRT-PCR results using STEM (Short Term Expression Miner) software [12]. STEM was designed specifically for time-series expression data and is therefore well-suited to clustering the developmental series represented by the stem and peel segments we analyzed.

**Figure 3:**
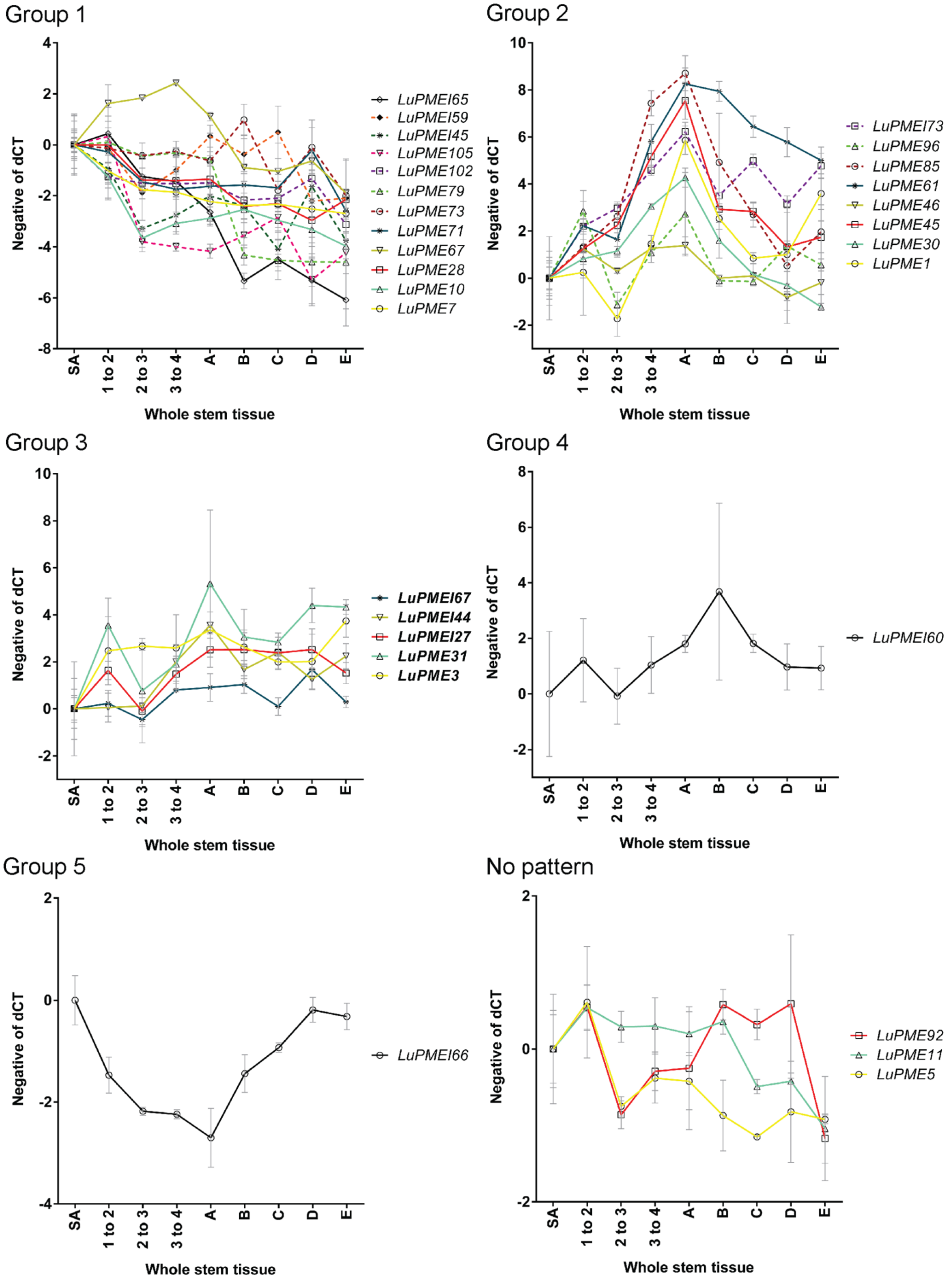
Clusters of transcript expression patterns in segments of whole stem tissues. Stems positions (SA, 1–2, 2–3, 3–4, A through E) are as defined in Figure 1. Transcript expression (y-axis) is the normalized negative dCT with point SA transformed to 0. Clusters are as defined by STEM software, using genes a minimum fold change of 4 between any two tissues.

Three genes for the whole stem tissues were filtered out and not used for the clustering because the difference in expression between the lowest value and the highest value was less than four-fold. Using STEM we identified five broad patterns among the transcript expression data from segments of the whole stem (Figure 3). In Group 1, which contained nine LuPMEs and three LuPMEIs, expression was highest in positions undergoing intrusive growth (SA though A), and decreased as the fibers matured (positions B through E). In Group 2, which contained seven LuPMEs and one LuPMEI, we observed an expression peak just above the snap point, at point A. In Group 3, which contained two LuPMEs and three LuPMEIs, expression was highest in positions below the snap point (B through E), which represent secondary cell wall deposition. In Group 4, which includes only one LuPMEI, peak expression occurred at point B. Finally in Group 5, one LuPMEI showed its lowest transcript expression at point A.

**Figure 4:**
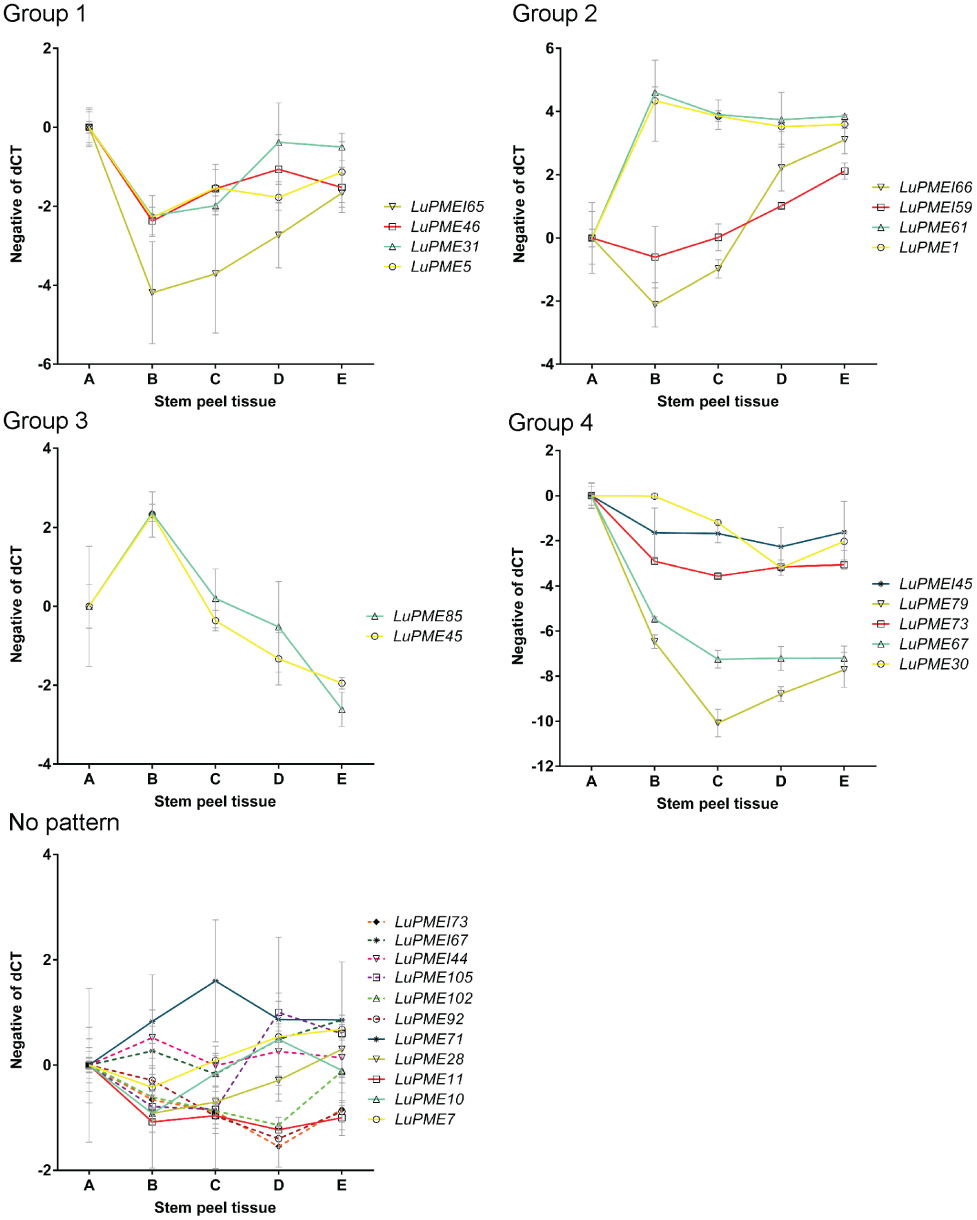
Clusters of transcript expression patterns in segments of stem peels. Stems positions (A through E) are as defined in Figure 1. Transcript expression (y-axis) is the normalized negative dCT with point SA transformed to 0. Clusters are as defined by STEM software, using genes a minimum fold change of 4 between any two tissues.

We also applied the same clustering method to transcript expression data from the outer stem peels. We eliminated 11 genes from clustering as the difference between the minimum and maximum dCT value was less than 2. Four different patterns were established (Figure 4). In Group 1, which contained four LuPMEs and one LuPMEI, a peak in expression was observed at point A (representing intrusive growth). In Group 2, which contained two LuPMEs and two LuPMEIs, transcript abundance generally increased as the fiber matured. In Group 3, which contained two LuPMEs, peak in expression occurred in position B, which was associated with the onset of secondary cell wall thickening, and expression decreased rapidly below this point. Group 4 contained three LuPMEs and one PMEI, and showed an expression minimum at point B, when secondary cell wall deposition started, and then the expression increased basally (points C, D, and E) as the fibers matured.

To assess the statistical significance of the differences between tissues in a given gene, we performed an ANOVA statistical analysis for the expression of the genes in the whole stem and the stem peel tissues, which is depicted in Tables S3 and S4, respectively.

### The PME activity is lower at the top of the plant

PME activity in the nine different segments of whole stem and five segments of stem peels was assessed, using three biological replicates, which were each measured in three technical replicates. In this assay, proteins extracted from stem segments were allowed to radially diffuse from a well into an agarose gel containing pectin and ruthenium red, and PME activity was detected by the development of a dark halo around the well. Measurement of the area of the halo allowed for a semi-quantitative estimate of PME activity.

We used standard curves with different concentrations of proteins extracted from a flax stem and pectinesterase from orange peel (Sigma) to determine if the area of the halo is directly proportional with the concentration of protein. We found that the area of the halo was positively correlated with PME concentration (*R* ^2^ = 0.96 for proteins extracted from flax and *R* ^2^ = 0.94 for the commercial PME; Figure S2), which supports the use of the radial assay to quantify the activity of the PMEs.

**Figure 5:**
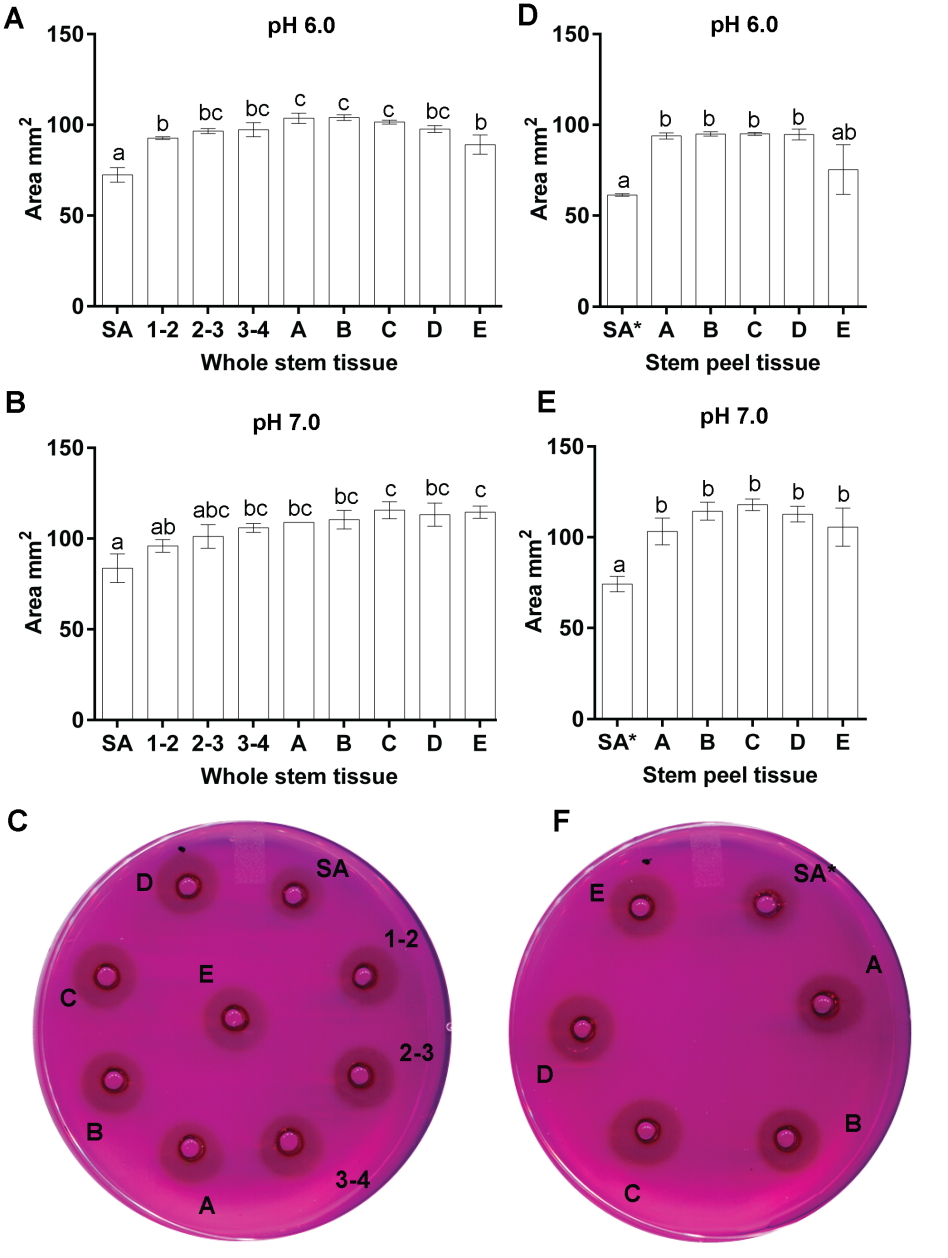
PME activity of native flax proteins. Proteins extracted from whole stems or stem peels at different developmental stages (as defined in Figure 1) were assayed for PME activity using a radial diffusion assay. In this semi-quantitative assay, the area of the halo formed was proportional to PME activity. The bar graphs show results of an ANOVA followed by Tukey’s multiple comparisons test from three technical replicates of each of three biological replicates. The plates show results of a representative diffusion assay at pH 7.0. Panels A to C correspond to the activity of proteins extracted from the whole stem. Panels D to F correspond to proteins extracted from the stem peel, plus stage SA of whole stem (SA*), for comparison purposes.

We assayed PME activity at both pH 7.0 and pH 6.0. These pH values were chosen based on the results of a pilot study of flax stem PME activity at pHs 5.0, 6.0, 7.0 and 8.0, which showed maximum activity at pH was 7.0 (data not shown). We also conducted the full assay at pH 6.0, since this was representative of the pH of the natural cell wall. In whole stem tissues at either pH 6.0 or 7.0, PME activity was significantly lower (p<0.05) at position SA relative to almost all other tissues (Figure 5, Table S5). This was also true in the stem peel, where PME activity was lower in SA than in any other tissues tested. Furthermore, the activity of SA (whole stem) was significantly lower (p<0.05) compared to the stages A to E of the stem peel tissues (Figure 5 panels D to F). Thus, PME activity (as a proportion of the total proteins extracted) appears to be highest in tissues below the apical-most 1 cm of the stem, with a peak around position A, and is higher in stem peels than in whole stem.

### Heterologous expression

LuPMEI45, whose transcript abundance peaked in expression during intrusive growth and diminished towards the bottom of the stem was selected for heterologous expression in *E. coli.* Furthermore, LuPMEI45 was chosen because it was one of the LuPMEIs, together with LuPMEI65, that showed a significant enrichment in expression during intrusive growth in the stem peel tissues (Figure 4, Table S4).

Three different strains of *E. coli* were tested for their suitability in heterologous expression of LuPMEI45: BL21(DE3), Rosetta(DE3)pLysS, and Rosetta-Gami B(DE3)pLysS (Novagen, Madison, WI, USA). We also evaluated different IPTG inducer concentrations (0.5 and 1 mM), induction times (2 hours, 4 hours, 18 hours), and induction temperatures (20°C, 30°C, and 37°C), and purification methods. We found that 1 mM IPTG, and 18 hours of induction at 20°C, were the best parameters for induction (data not shown).

LuPMEI45 expression was successfully detected in all of the strains, but the concentration was highest in Rosetta-Gami B(DE3)pLysS, so this strain was used in further experiments. The His-tagged heterologous LuPMEI45 protein was partially purified, and its identity was confirmed by LC MS/MS (Table S6) analysis and assayed in a radial diffusion assay. The recombinant LuPMEI45 protein was not purified to homogeneity and therefore the extract still contained some residual *E. coli* protein (Figure S3). Therefore an empty pET22b(+) vector expressed under the same conditions in Rosetta-Gami B(DE3)pLysS was used as a negative control in subsequent functional assays.

**Figure 6:**
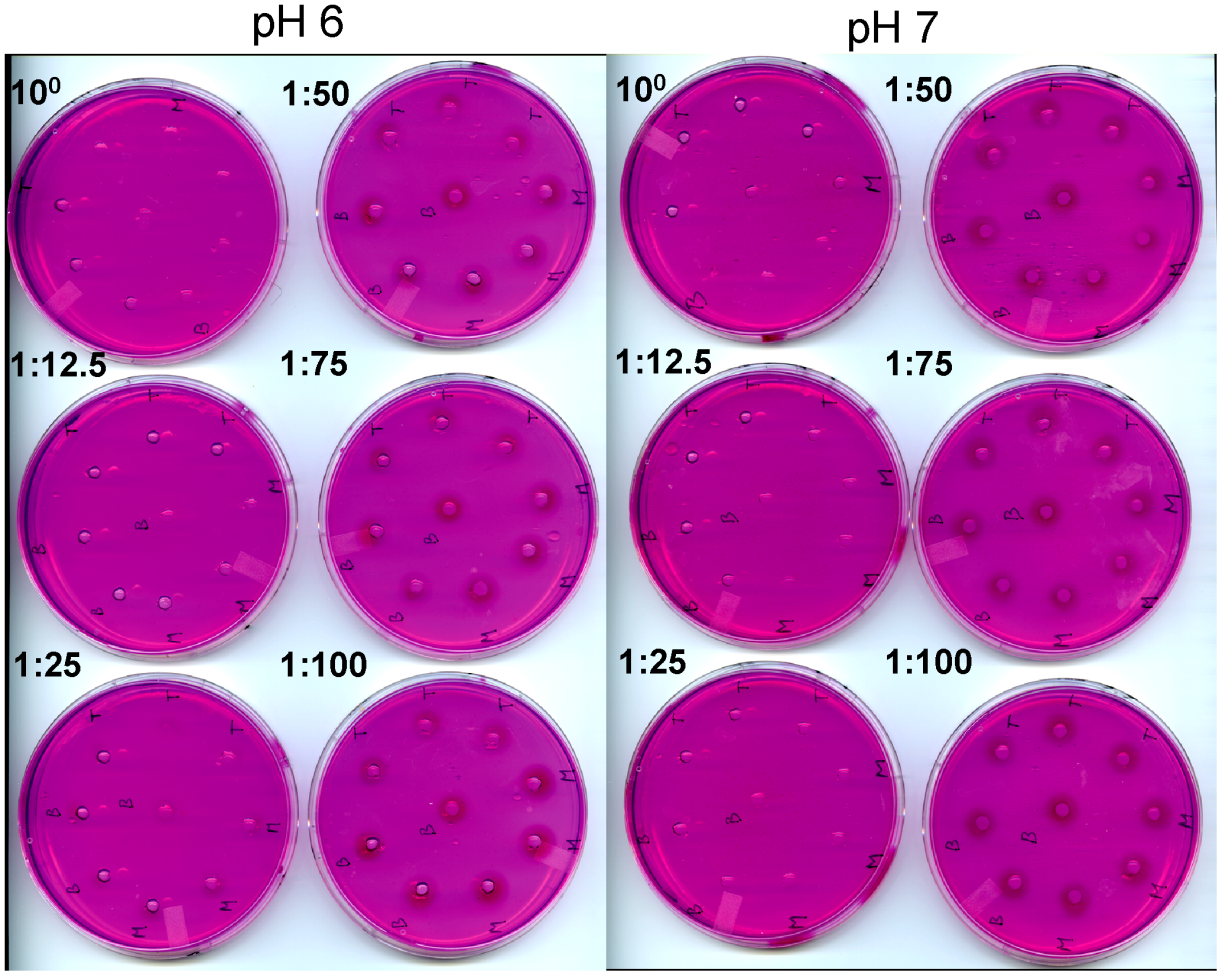
Radial assay of inhibitory capacity of LuPMEI45. Different dilutions of the purified proteins (7310 µg/mL) were assessed to establish the concentration at which ∼50% of the PME activity (396 µg/mL of flax proteins) was inhibited. The letters in the plates denote the position of the stem were the proteins were extracted: Bottom (B), Medium (M), and Top (T).

We found that recombinant LuPMEI45 successfully inhibited native PME activity of flax stem protein extracts, while no inhibitory activity was observed from the proteins extracted from the vector control or the dialysis buffer (Figure S4). The purified protein at a concentration of 7310 µg/mL was diluted at 1∶12.5, 1∶25, 1∶50, 1∶75, and 1∶100. We tested volumes of 10 µL of the different dilutions against proteins extracted from the top of the stem (first 5 cm), middle (11 to 16 cm from apex), and bottom (40 to 45 cm from apex), all at a concentration of 396 µg/mL (10 µL added). We determined that at both pH 6.0 and pH 7.0, a 1∶50 dilution, 146 µg/mL of LuPMEI45, was sufficient to reduce native LuPMEs activity by approximately 50%, while a 1∶12.5 dilution, 585 µg/mL, was sufficient to achieve a 100% inhibition in all the tissues (Figure 6).

**Figure 7:**
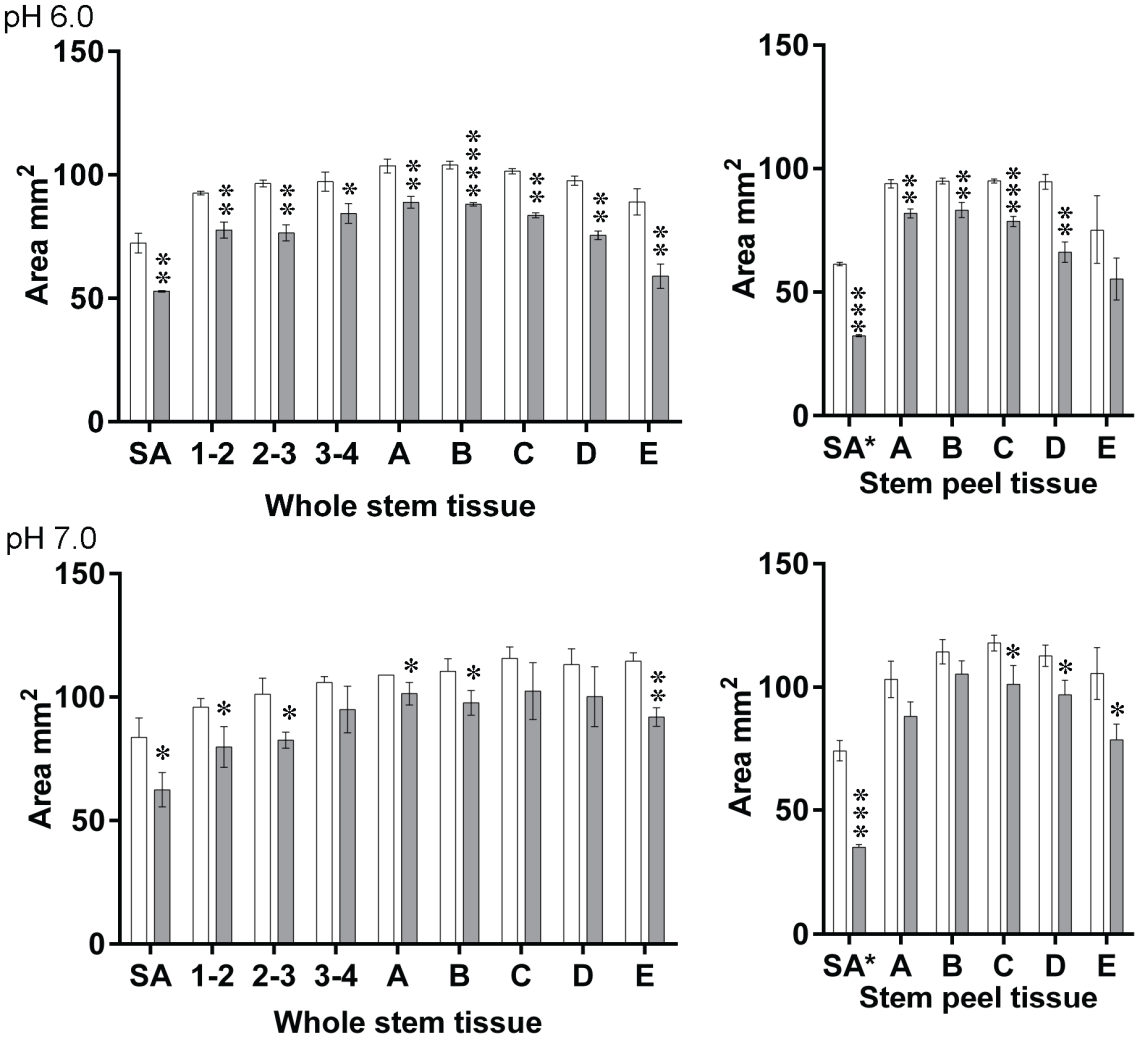
LuPMEI45 inhibitory activity on flax proteins extracted from whole stem and cortical peel. 10 µL of proteins extracted from the different tissues (396 µg/mL) were mixed with 10 µL of LuPMEI45 (146 µg/mL) (grey filled bars) or with 10 µL of the buffer (empty bars) in which LuPMEI45 was dialyzed. A t-test was done to determine if the activity of LuPMEI45 significantly reduce the PME activity at the different tissues. The asterisk denotes the p-value as follows. *0.01–0.05; **0.001–0.01, ***0.0001–0.001; ****<0.0001.

Once we knew the necessary concentration of heterologous LuPMEI45 to inhibit ∼50% of the PME activity, we expanded the assessment of the inhibition capacity of LuPMEI45 to cell wall proteins extracted from the nine different points in the whole stem and 5 different points in the stem peel used along this study (Figure 1). It showed significant inhibition (p<0.05) at pH 6.0 at all the points in the whole stem, and all, except point E, in the stem peel, and at pH 7.0 it inhibited at points SA, 1–2, 2–3, A, B, and E from the whole stem, and at points C, D and E form the stem peel, the activity of the PMEI on the whole stem SA in the stem peel tissues is shown as a reference (Figure 7).

## Discussion

To identify PMEs and PMEIs that were expressed dynamically during fiber development, we calculated the maximum fold difference in transcript abundance for any two tissues set, for each gene assayed (Table 1). This was done separately for both whole stems and stem peels. We also calculated the fold difference in transcript expression at equivalent positions in whole stems and stem peels (Table 2). If a gene was important for fiber development, the magnitude of enrichment was expected to be at least as high in stem peels as it was in whole stems. On the other hand, if a pattern was only observed in the whole stem, or if the magnitude of the change was significantly higher in the whole stem than in the stem peel, then that gene could be rather implicated in xylem development.

### Genes enriched in fiber containing tissues during fiber elongation

All the genes that had a similar pattern of transcript expression in the whole stem and the stem peel showed enrichment during intrusive growth. As the fold change was of at least the same magnitude in the stem peel compared to the whole stem, this meant that the expression of these genes may be specific to fibers and surrounding tissues. Those genes were *LuPME46*, *LuPME67*, *LuPME73*, *LuPME79*, *LuPMEI45*, and *LuPMEI65* (Figures 3 and 4). Here we will discuss the genes that are most likely to have important roles in fiber development, based on the magnitude of their transcript enrichment. We note that transcript abundance is not necessarily correlated with protein abundance or ultimate expression and activity, and that this important limitation must be considered when interpreting the data we present here.

*LuPME67* and *LuPME79* showed the largest change between the lowest and highest dCT values in the stem peel (152 and 1082 fold respectively, Figure 3) and a comparatively low change in the whole stem (20 and 26 fold respectively), which is evidence of fiber-specific enrichment of these genes. *LuPME67* expression drastically diminished (p<0.05) below the snap point in stem peel tissues, in relation to point A (Figure 4, Table S3). Based on the whole stem results, it can be concluded that the expression was constant above the snap point (p>0.05), only presenting a difference between points SA to 3–4, where 3–4 was significantly larger (p<0.05) (Table S3), which might indicate that as fibers increased in number in a given section [2], the gene expression also increased. *LuPME67* is a type 2 PME, and one of the few LuPMEs with a predicted acidic isoelectric point (pI 5.63), which implies that its mode of action might be random, leading to cell wall loosening as the pectin becomes a substrate for polygalacturonases and pectate lyases [19], [20]. Consequently, this is a gene that can be inferred to be active in the dissolution of the middle lamella between cells that the fibre penetrates during intrusive growth.

*LuPME79* showed a drastic decrease in expression below the snap point, and its expression was constant above the snap point, there was no difference (p>0.05) detected between stages of development SA to A in whole stem tissue (Table S3). *LuPME79* is a type 1 PME, which interestingly does not have a predicted cleavage for separation of the PMEI-like domain from the PME domain [3]. It has a basic isoelectric point (pI predicted 9.04), which indicates *LuPME79* may demethylesterify the homogalacturonan (HG) in a blockwise fashion [19], leading to calcium cross linking between HG domains [21], and ultimately to cell wall stiffening. It has been shown that type 1 PMEs are retained in the Golgi until the pro-region is cleaved out by subtilisin proteases [22] which are co-expressed with the PMEs [23]. However, it was shown that *LuPME3* can be secreted to the cell wall without processing the pro-region [24], so *LuPME79* may likewise be secreted without processing. The persistence of the pro-region (PMEI-like domain) may affect the PME activity, of this protein, so it will be informative to achieve its heterologous expression in the future.

*LuPMEI45* and *LuPMEI65* were the LuPMEIs found to have similar patterns in both the stem peel and in whole stem tissues; they both had significantly higher expression in stem peel tissues (p<0.05) in point A as compared to the tissues below the snap point, meaning that they are genes involved in the regulation of LuPMEs expression in the stem peel above the snap point. *LuPMEI45* was chosen for heterologous expression.

The expression of *LuPME5*, a type-1 PME with a predicted pI of 9.53, did not display major changes among the tissues in the whole stem (less than four-fold change), however, in the stem peel we did observe a higher expression in A, than in B, C, and D (p<0.05), although the largest fold change was only 5-fold. This is consistent with observations of Al-Qsous and collaborators [25], who determined that its highest expression occurs in the elongating parts of the hypocotyl, the apex and the root tip.

### Genes enriched in fiber-containing tissues below the snap point

From the stem peel expression data, we identified two genes that showed increased expression below the snap point (points B to E) respect to A (p<0.05) and their expression was stable between points B to E (p>0.05). *LuPME1* had a 20-fold change and *LuPME61* had a 24-fold change, between the minimum point (A) and the maximum point (B). The expression pattern of these genes in the stem peel was not similar to their expression in the whole stem, in which the expression, oppositely, diminished from A to B in *LuPME1*, and did not change from A to B in *LuPME61*. This means that the expression observed in the stem peel for these genes is a specific to this tissue, and indeed expression of *LuPME1* was 53, 121, and 86 times higher in points B, C, and D, respectively, in stem peels as compared to whole stem tissues (Table 1). As described above, the mode of action of these genes might be blockwise demethylesterification, so they would aid in the strengthening of the cell wall once the cells stop elongating (below the snap point) [2]. The role suggested for these genes is based on analysis of an orthologous gene from Arabidopsis, AtPME35 [3], which was found to strengthen the inflorescence stem by a blockwise demethylesterification action [13].

### Genes enriched in the xylem

We found seven genes that showed a peak in expression in point A of the stem. These included the four genes that showed the highest fold change (between any two stem points) among any of the genes analyzed in the whole stem tissue: *LuPME85* (419-fold), *LuPME61* (306-fold), *LuPME1* (191-fold), and *LuPME45* (186-fold). The other three genes were *LuPME30* (45-fold), *LuPME31* (40-fold), and *LuPME96* (16-fold) (Table 1). As this expression pattern was unique for the whole stem and was not observed in the stem peel tissues, we concluded that these genes may play a role in xylem or pith development. The predicted isoelectric point of all of these proteins is basic, so blockwise demethylesterification is expected to occur [19] leading to cell wall rigidification. Furthermore, one PMEI, *LuPMEI73*, was observed with this pattern in the whole stem but not in the stem peel; its high expression in point A, respect to the SA (74-fold), leads us to speculate that it has an important role in regulating PME activities at this point in the inner tissues, presumably within the xylem.

*LuPME3* (type-1 PME with a predicted pI of 9.8) expression was not detected in the stem peel, while it was detected in low amounts in the whole stem tissues where its expression was significantly lower in SA (p<0.05) with respect to the rest of the tissues (1–2 to E), where the expression was not significantly different (p>0.05). The xylem undergoes differentiation, expansion, and maturation. In the vicinity of the shoot apex, very little vascular tissue maturation is expected to occur and it is only at node 3–5 that thickening starts [26], so if *LuPME3* is involved in the cell wall stiffening of the xylem, it is then expected that its expression is lower in point SA, which we found, and then as more xylem is produced along the stem the maturation of the xylem is a constant process which is observed in the expression of this gene. *LuPME3* was previously found to have detectable expression in the vascular tissue of stems and leaves, and in the root meristem [27], and was found to have similar expression in the whole extension of the hypocotyl and the root in a 10 days old seedling [25]; they did not find lower expression at the top of the seedling, however, their detection method (RT–PCR Southern blot) is not as sensitive as qRT-PCR. Based on the phylogenetic analysis done by Pinzon-Latorre and Deyholos [3], it was established that *LuPME3* is one of the most similar genes to *PttPME1* [3], a PME in hybrid aspen that Siedlecka and collaborators [28] determined that when it was downregulated the xylem fibers elongation increased, so it was suggested that PttPME1 strengthens cellular adhesion, hindering intrusive growth. As the expression of *LuPME3* in flax occurs in the xylem, it is possible that the same situation is occuring in flax.

*LuPME7* and *LuPME92* are the other LuPMEs closely related to *PttPME1*. The expression of *LuPME7* was significantly higher (p<0.05) in the whole stem in point SA respect to A, B, C, D, and E (Table S3), while in the stem peel there was no significant difference between the tissues (Table S4). *LuPME92* expression did not show a difference higher than 4-fold between any of the tissues in the whole stem and the stem peel, however the expression was higher in the whole stem tissues (A to E). With respect to stem peel, point D was 26 times higher in the whole stem than in stem peel (Table 2), suggesting a role in xylem maturation, as the one observed for PttPME1 [28].

### PMEI inhibitory activity

LuPMEI45 was found to effectively inhibit the action of flax LuPMEs along the stem (Figure S4). As the expression of LuPMEI45 is higher during intrusive growth (p<0.05) (Tables S3 and S4), it could be expected that its inhibitory capacity is higher at the tissues undergoing intrusive growth, however the inhibitory capacity was not significantly different along the stem (data not shown). The preferred target(s) of LuPMEI45 will be important to determine, so its activity can be correlated with the mode of action of a PME.

## Conclusion

We were able to characterize in detail the transcript expression of selected LuPMEs and LuPMEIs along the stem, in relation to stages of development of flax fibers. Candidate genes with expression patterns involving them in specific processes of phloem fibers and xylem development were presented, and a functional heterologous expression of one of them was achieved. The detailed study of these genes by the subcellular localization of the proteins, mutagenesis, silencing and/or overexpression techniques will allow the finding of genes relevant for the improvement of the crop, either by producing longer and easier to extract fibers or by obtaining plants with shorter fibers avoiding the obstruction of the machinery.

## Supporting Information

Figure S1 **Transcript expression of genes from whole stem and stem peel tissues (dCT).** dCT was obtained by subtracting the geometric mean of the three endogenous controls used to the Ct value of the genes studied for every biological replicate. Here we show the average of the three biological replicates. The tissues below the dotted line are whole stem tissues, and below the solid line are stem peel tissues.
(TIF)

Figure S2 **Standard curve of PME activity by radial assay.** Proteins extracted from the whole stem (a) or pectinesterase from orange peel (b) were used at different concentration in a radial assay to assess the correlation with the area of the halo they produced.
(TIF)

Figure S3 **Purification of LuPMEI45 expressed in** ***E. coli.*** *Excised band of LuPMEI45 (∼ 20.7 KDa) successfully identified by LC MS/MS analysis. Left: Protein ladder. FT: Flow through; W: Wash; E: Elution. W1∶50 mM Tris-HCl 1.5 M NaCl. W2∶50 mM Tris-HCl, 300 mM NaCl, 20 mM Imidazole. W3∶50 mM Tris-HCl, 300 mM NaCl, 40 mM Imidazole. E: 50 mM Tris HCl, 1 M NaCl and 250 mM Imidazole
(TIF)

Figure S4 **Inhibitory capacity of LuPMEI45 expressed in** ***E. coli.*** The activity of LuPMEI45 was assessed in a radial assay measured as its capacity of blocking the activity of flax cell wall proteins extracted from the top 5 cm of a ∼5 weeks old plant. Two different controls were used: The buffer used for the dialysis of the protein after purification, and the purified proteins from the empty vector, pET22b(+), expressed in the same system under the same conditions.
(TIF)

Table S1 **Selected LuPMEs and LuPMEIs for expression profiling at different fiber developmental stages.**
(XLSX)

Table S2 **Significance of the difference on the expression of the genes in the same point of the stem in stem peels and whole stem tissues.** Statistical significance was determined using the Holm-Sidak method, correcting for multiple comparisons. The asterisks denote the significance of the test based on the p-value as follows. *0.01–0.05; **0.001–0.01, ***0.0001–0.001; ****<0.0001. ns: non-significant difference (p>0.05). np: no pattern.
(XLSX)

Table S3 **Tukey’s multiple comparisons test of dCT expression values between whole stem tissues.** An ANOVA test was followed by a Tukey’s multiple comparisons test using GraphPad Prism version 6.00 for Windows The asterisks denote the p-value as follows. *0.01–0.05; **0.001–0.01, ***0.0001–0.001; ****<0.0001. ns: non-significant difference (p>0.05). np: no pattern.
(XLSX)

Table S4 **Tukey’s multiple comparisons test of dCT expression values between stem peel tissues.** An ANOVA test was followed by a Tukey’s multiple comparisons test using GraphPad Prism version 6.00 for Windows. The asterisks denote the p-value as follows. *0.01–0.05; **0.001–0.01, ***0.0001–0.001; ****<0.0001. ns: non-significant difference (p>0.05). np: no pattern.
(XLSX)

Table S5 **Tukey’s multiple comparisons test of Pectin Methylesterase activity along the flax stem.** An ANOVA test was followed by a Tukey’s multiple comparisons test using GraphPad Prism version 6.00 for Windows. SA*: SA tissue from whole stem compared to stempeel tissue. The asterisks denote the p-value as follows. *0.01–0.05; **0.001–0.01, ***0.0001–0.001; ****<0.0001. ns: non-significant difference (p>0.05).
(XLSX)

Table S6 **LC MS/MS analysis results of excised band at the expected size for LuPMEI45.** A coverage of 57.06 was obtained. 305 peptide spectrum matches (PSMs) corresponding to 9 unique peptides matching LuPMEI45 were identified.
(XLSX)

File S1 **Codon optimized sequence of LuPMEI45 expressed in** ***E. coli.***
(DOC)
