# Supplementary figures and images for: Pectinmethylesterases (PME) and Pectinmethylesterase Inhibitors (PMEI) Enriched during Phloem Fiber Development in Flax (Linum usitatissimum)
Source: PLoS One. 2014 Aug 14;9(8):e105386. doi: 10.1371/journal.pone.0105386 (PMC4133374; doi:10.1371/journal.pone.0105386)

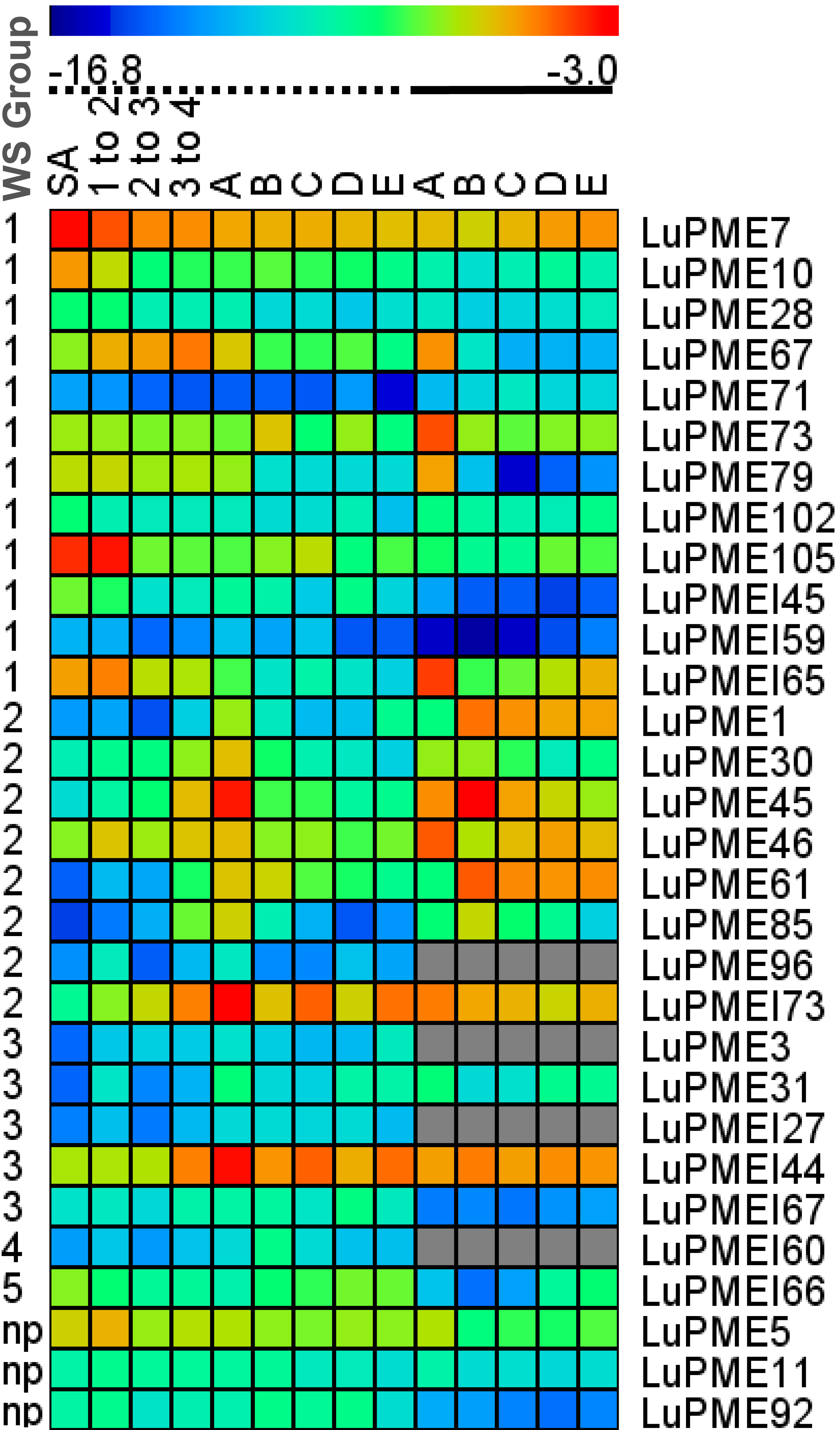

Supplement: Figure S1 — Transcript expression of genes from whole stem and stem peel tissues (dCT). dCT was obtained by subtracting the geometric mean of the three endogenous controls used to the Ct value of the genes studied for every biological replicate. Here we show the average of the three biological replicates. The tissues below the dotted line are whole stem tissues, and below the solid line are stem peel tissues. (TIF) [file pone.0105386.s001.tif]

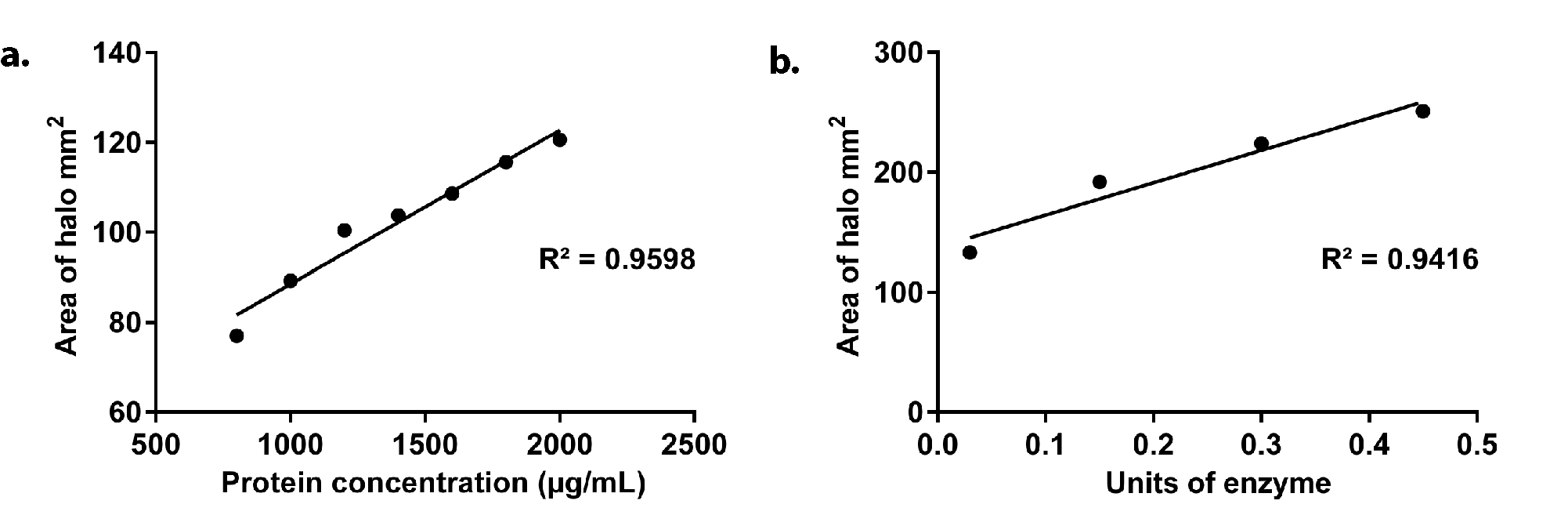

Supplement: Figure S2 — Standard curve of PME activity by radial assay. Proteins extracted from the whole stem (a) or pectinesterase from orange peel (b) were used at different concentration in a radial assay to assess the correlation with the area of the halo they produced. (TIF) [file pone.0105386.s002.tif]

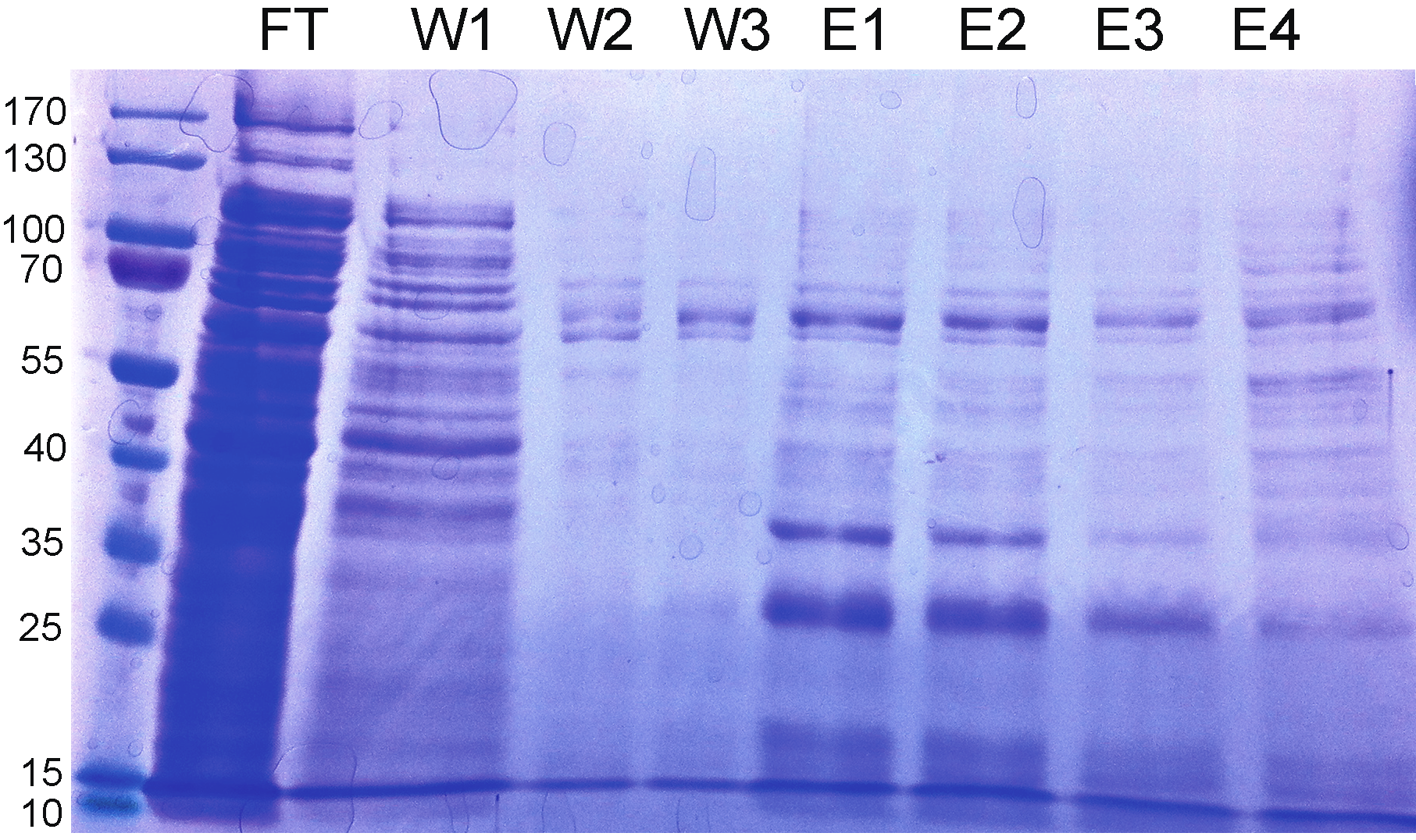

Supplement: Figure S3 — Purification of LuPMEI45 expressed in E. coli. *Excised band of LuPMEI45 (∼ 20.7 KDa) successfully identified by LC MS/MS analysis. Left: Protein ladder. FT: Flow through; W: Wash; E: Elution. W1∶50 mM Tris-HCl 1.5 M NaCl. W2∶50 mM Tris-HCl, 300 mM NaCl, 20 mM Imidazole. W3∶50 mM Tris-HCl, 300 mM NaCl, 40 mM Imidazole. E: 50 mM Tris HCl, 1 M NaCl and 250 mM Imidazole (TIF) [file pone.0105386.s003.tif]

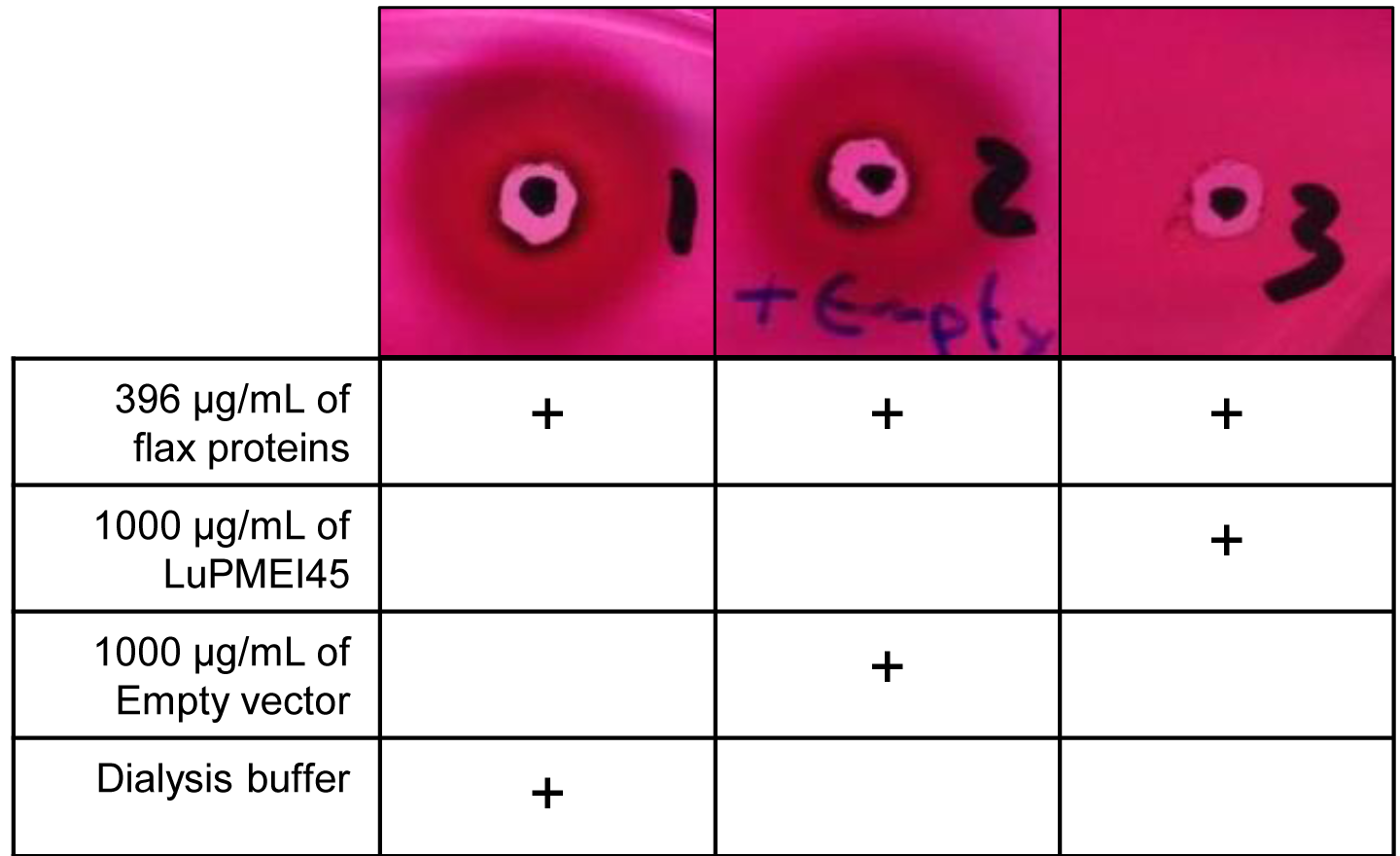

Supplement: Figure S4 — Inhibitory capacity of LuPMEI45 expressed in E. coli. The activity of LuPMEI45 was assessed in a radial assay measured as its capacity of blocking the activity of flax cell wall proteins extracted from the top 5 cm of a ∼5 weeks old plant. Two different controls were used: The buffer used for the dialysis of the protein after purification, and the purified proteins from the empty vector, pET22b(+), expressed in the same system under the same conditions. (TIF) [file pone.0105386.s004.tif]
