# Supplementary material for: Pectinmethylesterases (PME) and Pectinmethylesterase Inhibitors (PMEI) Enriched during Phloem Fiber Development in Flax (Linum usitatissimum)
Source: PLoS One. 2014 Aug 14;9(8):e105386. doi: 10.1371/journal.pone.0105386 (PMC4133374; doi:10.1371/journal.pone.0105386)
Supplement: File S1 — Codon optimized sequence of LuPMEI45 expressed in E. coli. (DOC) [file pone.0105386.s011.doc]

>LuPMEI45_no-signal-peptide_codon-optimized

ATGGCCGACACCGACTATATCCAAACTTCTTGCCAGGCGTCCACCCGTTACCCGGATCTGTGTATCTCTACCCTGTCTCCGCAGGCTTCTAACATTACCACTCCAAAACTGCTGGCCTCTGCGGCTCTGTACGCCGCTCTGGCAGCGGCAAAATCCACCTCCAAAAGCATTGAAACCCGTCCTTCCTCTTGGAGCTCTCGTCTGCGCGATTGTCGCGAAGAGATGAGCGACAGCGTTGACCGTCTGCGTGATTCCGCGAAAGAAATGAAGGGTGAAGTCGTTCTGTCCCGTTTCCAGGTAAGCAACGTGCAGACGTGGGCTTCCGCAGCAATGACTTGCATGGACACCTGCACGGATGGTCTGGTGGAAGGTGAAGTAAAACGCTGGGTCGTTGAACGTTCTGGTATCGTTAAGGCGGGCTTCCTGATCAGCAACGCGCTGGCTTTTGTTAATAAATACGGCGATGGCCTGGTGAACCAG
